# Health Status, Health-Related Factors and Work Environment in Korean Semiconductor Workers between 1984–2012: A Qualitative Study and a Cross-Sectional Study

**DOI:** 10.3390/ijerph19106286

**Published:** 2022-05-22

**Authors:** Kyungsik Kim, Ho Kyung Sung, Jieun Jang, Eunyoung E. Suh, Kwan Lee, Sue K. Park

**Affiliations:** 1 Department of Preventive Medicine, Seoul National University College of Medicine, Daehak-ro 101, Jongno-gu, Seoul 03080, Korea; kks6235@snu.ac.kr (K.K.); yanghyo25@snu.ac.kr (J.J.); 2 Department of Biomedical Science, Seoul National University Graduate School, Daehak-ro 101, Jongno-gu, Seoul 03080, Korea; 3 Cancer Research Institute, Seoul National University College of Medicine, Daehak-ro 101, Jongno-gu, Seoul 03080, Korea; 4 Institute for Public Healthcare, National Medical Center, Eulji-ro 245, Jung-gu, Seoul 04564, Korea; hokyungsung@nmc.or.kr; 5 National Emergency Medical Center, National Medical Center, Eulji-ro 245, Jung-gu, Seoul 04564, Korea; 6 Gyeongnam Center for Infectious Disease Control and Prevention, Jungang-daero 300, Uichang-gu, Changwon 51154, Korea; 7 College of Nursing Research Institute of Nursing Science, Seoul National University, Daehak-ro 101, Jongno-gu, Seoul 03080, Korea; esuh@snu.ac.kr; 8 Department of Preventive Medicine, Dongkuk University College of Medicine, Gyeongju 38066, Korea; kwaniya@dongguk.ac.kr; 9 Integrated Major in Innovative Medical Science, Seoul National University College of Medicine, Daehak-ro 101, Jongno-gu, Seoul 03080, Korea

**Keywords:** semiconductor workers, qualitative study, cross-sectional study, work environment, health-related factors, job-exposure matrix

## Abstract

Background: The environment of semiconductor facilities and exposure status has undergone changes. To identify changes in the work environment, health status, and risk factors, a qualitative and cross-sectional study was conducted. Methods: For the qualitative study, 38 current and retired workers who worked for at least 10-years were studied; for the cross-sectional study, 306 current workers who worked for at least 5-years based on JEM strata from Samsung Electronics were selected. Participants were asked about occupational history, cancer-relating risk factors during the working period, medical history, dietary habits for the past year, and hematological cancer-relating infection. Results: In the qualitative study, fabrication workers reported bladder cystitis, dermatitis in hands, gastritis/ulcer, and dry eye as frequent symptoms during work environment changes (manual to automatic process). In the cross-sectional study, there were no abnormal findings for infection biomarkers related to hematological cancer and spontaneous abortion, and their general health status was no different from the general population. Also, questionnaire feasibility was evaluated for the applicability in the prospective cohort. Conclusion: Current semiconductor workers’ health status was good compared to other populations. For lifelong health assessment, a cohort study is needed which considers health worker effects and current environments.

## 1. Introduction

In the 1980s, as an occupational disease, the risk of spontaneous abortion among semiconductor workers was first reported [1]. Since then, research about workers’ health problems, such as cancers, reproductive hazards, etc., and hazardous substances or detailed semiconductor processes that cause health effects in workplaces have been published [2,3,4,5,6,7]. However, the causal relationship between the risk of cancer and specific hazardous factors or semiconductor processes has not yet been assessed.

In June 2007, a female worker who was employed at a Samsung Electronics semiconductor facility died of leukemia, and five additional similar cases were reported where workers and their bereaved families initiated work-related lawsuits. They were all people who worked during the 1990s and 2000s. Consequently, the Korea Labor Welfare Corporation, the Ministry of Labor requested an epidemiological investigation for the evaluation of work-relatedness to the Occupational Safety and Health Research Institute (OSHRI) and Korea Occupational Safety and Health Agency (KOSHA). In 2019, an Epidemiological investigation from the OSHRI showed that the hematopoietic cancer of workers was related to semiconductor works but needed to be interpreted carefully due to the detection bias and the low number of rare disease cases [8]. Along with this, debate persisted about the causal relationship between cancer and the semiconductor work or occupational factors [9,10,11]. At that time, a Samsung Ombudsperson Commission was launched to inspect Samsung Electronics’ internal disaster management system and initiate an improvement of the semiconductor environment [12]. Additionally, in 2018, the Samsung Electronics Semiconductor, LCD industrial Health Support Compensation Committee was established to compensate workers for diseases including leukemia [13]. In addition to these efforts, it is necessary to conduct an exposure assessment from the past to the present for semiconductor workers’ health effects monitoring.

However, the current semiconductor work environment may be different now, compared to the past, so it is necessary to understand the environment changes from the past to the present. Thus, a qualitative study was conducted based on the current and retired workers whose employment period was higher than 10 years in a focus group interview. Additionally, we conducted a cross-sectional study to evaluate the health status and possibly related factors in the current semiconductor works. In the cross-sectional study, both questionnaire and the infection status (helicobacter pylori, hepatitis B virus, hepatitis C virus, human immunodeficiency virus type 1 (HIV 1), and Ebstein-Barr virus (EBV) were assessed according to the semiconductor job categories. Finally, in advance of conducting a prospective cohort study on the overall semiconductor workers, this study tried to verify the usefulness of the questionnaire tool.

## 2. Materials and Methods

### 2.1. Qualitative Study: Focus-Group Interview

#### Collection of Information in the Qualitative Study

In advance of the focus group interview, participants were asked to fill out a self-reported questionnaire about their occupational histories such as processes, occupational substance use, and common diseases in each group. The main questions were: (1) What was the environment in which workers worked in the past? How is it different from the present? (2) Have you ever been exposed to odors, air quality, or environmental exposure substances in the past and present working environments? (3) How was your shift work? (Please explain if it is different from what is indicated on the questionnaire). Isn’t shift work difficult? (4) If there have been any clusters of symptoms or diseases that have occurred to you and your co-workers from the past to the present, please tell me as much as you can remember. For the interview, each group and interviewer attended the meeting and talked freely for about 30 min to 1 h to answer the main questions. The interview contents were recorded with the consent of the participants, and the recorded contents were organized into scripts. The qualitative study analysis was conducted as terms first and then in phrases. After that, the data were analyzed according to each group. The details of the questions are described in Table S1.

### 2.2. Cross-Sectional Pilot Study

#### Collection of Information in the Cross-Sectional Study

To collect the information, semiconductor workers were asked to visit a separate place for the research. Because of their shift work patterns, research consent with a reported questionnaire was obtained one hour before their work time. All of the workers completed the questionnaire by themselves first, if there was any non-responded questions or they could not understand the question, the research assistant helped with the answer. If some questionnaires needed additional work, it was checked after the shift work. In addition, 15 mL of workers’ blood and urine were collected to assess their infection status along with an assessment of occupational substances. As collection items, the subcontractor workers collected urine only. As a detailed questionnaire item, occupational history including employment period, shift work pattern, fab work status, individual disease history including cancer, drug, medical radiation exposure status, lifestyle factor including smoking, drinking, and physical activity, and their dietary habits. Occupational history was reported by the semiconductor workers themselves, but detailed processes, job duty, and factory district were referred to in the current facilities records at the human resource department. In the case of personal disease history, the workers reported by themselves, but was based on the diagnosis of diseases at the medical center or the health examination center. For their dietary habits, we identified their food consumption by the food frequency questionnaire and their usual eating habits. The individual’s infection status was confirmed by hepatitis B virus (Chemiluminescent Microparticle Immunoassay), hepatitis C virus, HIV-1, H. Pylori (Enzyme-linked immunosorbent assay, ELISA), and EBV (Sandwich ELISA) based on the blood samples.

### 2.3. Statistical Analysis

We classified semiconductor workers into seven groups as follows: (1) Office work (men and women), (2) Assembly work (men and women); (3) Operators (women); (4) LCD workers (men); (5) Engineers working for non-implant semiconductor processes (men); (6) Engineers working for ion-implant (men) (Employed under Samsung Electronics); (7) PM engineers (ion-implant process employed in subcontractor workers). Detailed statistical analysis, overall, and seven groups were described using the mean, median for continuous variables, and proportion for categorical variables. Differences in distribution among the seven groups were tested using analysis of variance for continuous variables and chi-square test for categorical variables, and *p*-values for the differences were presented.

## 3. Results

In the process of the study population selection, both qualitative and cross-sectional studies were considered six basic criteria and constructed a group according to criteria to identify the overall and the eligible number of semiconductor workers. First, (1) semiconductor workers were classified as current, retired, and subcontractor workers. Then (2) identify each worker’s work district, and (3) identify non-fab, fab, LCD, and assembly work in large job categories. Next, (4) their detailed job duty and specific processes would classify and (5) divided the workers according to the gender. Lastly, (6) the group is divided according to their employment period into two (≥15 years or 10–14 years in the qualitative study) or three groups (≥15 years, 10–14 years, or 5–9 years in the cross-sectional study) (Figure S1).

Accordingly, the eligible subjects were extracted by stratified random sampling with unequal probabilities without replacement, then we asked workers if they agreed to participate as subjects in this study. In addition, the study population of the cross-sectional study was selected by considering workers’ shift work, and the ratio was 1:1:1 in each shift work Day (06:00–14:00), Swing (14:00–22:00), Graveyard (GY, 22:00–06:00). However, for the retired and subcontractor workers included in the qualitative study and subcontractor workers in the cross-section study, their overall and eligible subjects could not confirm. In the case of the retired workers, due to the personal information protection act in Korea, we could not get any personal information such as addresses or contact numbers. In the case of subcontractor workers, there was a social issue of companies laying down the law for the workers. Therefore, the semiconductor facility could not ask for detailed information from subcontractor workers directly. Thus, research consent was the primary role of the research participation. After that, only agreed subcontractor factories and their workers could include in the study population. According to the process, there were 4594 eligible subjects in the qualitative study, and 32 workers (0.7%) were randomly selected, and six retired and subcontractor workers were non-randomly included in the study population (38 participants). In the cross-sectional study, there were 8059 eligible subjects, 239 current workers (3% from Samsung Semiconductor Electronics population) were selected, and 67 (11.2% from the subcontractor population) out of 596 subcontractor workers were selected as the study population (306 participants). We described the detailed eligible subjects in each group in Tables S2 and S3.

There were 26 males and 12 females in the qualitative study aged in their 40s and their dialogues are described in Table S2. The participants stated that some processes required manual work in the past, while all current processes were automated so far. Based on their detailed statements, workers who worked in the 1980s, directly dealt with the volatile organic compounds (i.e., TCE) with their hands. However, it is known that TCE has not been used in the company since 1995. Additionally, workers in the past could be exposed to the surrounding chemicals because the workspaces were not separated according to their roles and processes in detail work. However, it was difficult to evaluate the possibility of intermittent high exposure, and other exposure environments (leakage or accident) could not be identified. What we confirmed was that 70–80% of chemicals were not detected and the air level of detected ones were less than 10% of the legal occupational exposure levels from 2016 to 2018. Therefore, there were few probabilities for exposure to environmentally harmful substances in the current working environment. In the past, workers were aware of chemical exposure by the smell of chemicals or the skin symptoms rather than information on the substances handled. Interviewees reported diseases during semiconductor work such as musculoskeletal pain, gastritis/ulcer, cystitis in the bladder, dermatosis in hands, dry eye and decreased vision, and lymphoma (in a beneficiary retiree).

In the case of the cross-sectional study, there were 220 males and 86 females (total 306 people), with a median age of 36 years (Table 1). As occupational characteristics, incumbent Samsung Semiconductor Electronics employees participated evenly according to the shift work (‘Day’, ‘Swing’, ‘GY’), but only 43% of the included PM engineers from the subcontractor workers do shift work. In the case of the working year, it was significant with semiconductors according to the groups, due to the short period in LCD and semiconductor processes workers (Table 1). Among the participants, 71% of the participants worked in the fab, but assembly workers and operators showed the lowest rate of fab work among the groups. As shift work, 3-shifts 4-group is the standard pattern now, but as the experience, they suffered shirt work pattern such as 2-shifts 3-group, 2-shifts 2-group (Table 1).

As individual lifestyle factors, we assessed smoking status, alcohol consumption and physical activity (Table 2). Current smoking rate, PM engineers (79%) responded the highest smoking rate among the study population compared to office workers (33%). But considered pack-year over 20 years, office workers (75%) showed a higher rate than other groups (below 22%) and it was statistically different (*p* < 0.05). Only men workers used e-cigarettes, which was higher in PM workers (18%) than office workers (4%) (*p* = 0.06). The lifetime drinking prevalence (the fraction of people who drink in the past and present) and the current drinking rate were 84% and 82%, respectively (Current drinking rates); and there was no significant difference in a lifetime and current drinking rates across the groups. Among the drinkers, the proportion of drinking more than 4 times per week was 22%, which was 32% and 27% in the non-implant group and PM engineers group, respectively, which was higher than that of the office workers (23%) (*p* < 0.05). In the case of physical activities, there was a difference between groups in both high and moderate intensity (*p* < 0.05), and the LCD and non-implant workers showed the highest rate among the groups.

The diseases consisted of cancers, rare diseases, and reproductive diseases. Similar to qualitative study, the most common diseases were gastritis or gastroduodenal ulcer (16%) and dermatosis (allergy/contact, 9%) in the cross-sectional study. In men, gastritis or gastroduodenal ulcer (11%) was the most common, and gastritis or gastroduodenal ulcer (20%), cystitis (14%), and dermatosis (14%) were observed in women. We hypothesized that family histories of diseases could be related to compensation and common diseases observed in workers, but no statistically significant difference was observed between groups. Gastroduodenal ulcer, dermatosis (allergic/contact), cystitis, and dry eye, could be classified as ‘Fabrication-relating diseases’, hypertension, hyperlipidemia, diabetes, angina pectoris, fatty liver, and liver disease (acute, chronic) were classified as cardiometabolic diseases. In the survey on personal medical history, 17% of the total subjects had multi-morbidity for two or more diseases. Across the working group, office workers had the highest multi-morbidity (38%), and the PM engineers and implant workers had the lowest multi-morbidity (7% and 5%), respectively (The difference across working groups, *p* = 0.07). The co-morbidity of two or more Fab-related diseases was highest among operators (23%), and the co-morbidity of two or more cardiometabolic diseases was highest among office workers (21%). Additionally, we described the detailed diseases list of compensation from Samsung Electronics in Table S4. There were differences between groups in multivitamin and ginseng intake (*p* < 0.05) (Table 3). Specific eating habits, there are differences between groups in skipping meals, eating alone, and coffee intake (*p* < 0.05) (Table 4). Additionally, no differences were observed between fabrication and non-fabrication groups or according to job categories for carcinogenic agents measured in semiconductor workers (Table 5). In the case of spontaneous abortion, there was no difference according to the job groups and only a limited number of events were identified (Table S5). In medical history, gastroenterography, computer tomography (CT), and magnetic resonance imaging (MRI) scan are significantly different according to job duty (*p* < 0.05).

## 4. Discussion

In this study, semiconductor workers were classified and selected according to the JEM. Also, we assessed changes in the semiconductor working environment from past to present based on a qualitative study. In addition, by the cross-sectional pilot study, long-term employment semiconductor workers were classified by JEM and assessed their occupational, lifestyle (alcohol consumption, smoking, physical activity), history and comorbidity of individual diseases, carcinogenic infection, and reproductive history. There was no difference between the groups, but some patterns were identified as semiconductor workers’ unique characteristics.

From the qualitative study, the health impact of the working environment was evaluated in both current and retired workers. Along with the change of the wafer size from the 1980s (4-inch) to 2000s (12-inch), the work environment had changed significantly due to fully automatic processes from some manual processes. Firstly, workers stated that TCEs were used directly as substances for cleaning or dissolving oil on their hands in the past. Second, due to the no spatial separation between processes, workers could be exposed the hazardous substance even they did not handle the material. However, it is difficult to generalize the situation to all workers, and there is a limit to reconstructing the actual exposure situation.

As is known, some diseases are reported mainly in semiconductor workers [14,15]. From the qualitative study, workers experienced musculoskeletal pain, gastritis or ulcer, cystitis in urinary bladder, dermatosis in hands, dry eye, and decreased vision which are classified as fab-relating diseases. In the pilot study, these diseases were observed more in fab workers and the highest in operators. Cystitis was reported in 14% of female workers and in 13% of operators. It may have been influenced by female anatomy (i.e., short urethra), hormonal changes due to pregnancy and menopause, and protective clothing worn in the fab. In the case of dermatitis, it may be due to the nature (i.e., material) of the protective clothing worn within the fab [16,17]. Therefore, improvements in the material, fit, the convenience of putting on and taking off, and problems around the female genitalia in a line should be considered in a way that does not become a problem for semiconductor errors. For example, as eye-related symptoms (i.e., dry eye syndrome, blurred vision) can be caused by the special lighting in the fab, especially around the photo process, protective equipment such as protective goggles should be considered.

The selected infectious agents are known to be associated with several cancers [18,19,20,21,22,23,24]. The infection rates of hepatitis B virus, hepatitis C virus and *H. pylori* in semiconductor workers were lower than those in Korean general population. The Korea National Health and Nutrition Examination Survey (KNHANES) in 2017 reported that the infection rates of hepatitis B virus (HBV) (measured by HBsAg) and hepatitis C virus (measured by anti-HCV antibody) were near 3% and 0.3–0.6% in people aged 30–49 years, respectively [25]. In contrast, the infection rates of HBV and HCV in semiconductor workers were 1% and 0%, respectively. The infection rates of *H. pylori* (measured by *H. pylori* IgG antibody) in Korean health examinees derived from 21 health screening centers were near 51% (male, 54.1%; female, 48.8%) [26], but those in semiconductor workers were 33%. In the case of hypertension, the prevalence rate in overall semiconductor workers was 8%. Each office and assembly workers showed 29% and 21%. Compared to the semiconductor workers, the hypertension prevalence of the general population in their overall 40s was 19.2% and it is similar to assembly workers but lower than office workers. In the case of diabetes, only assembly and LCD workers showed a prevalence rate of both 5% in the semiconductor workers. Compared to the general population in their 40s, it showed 6.9% and is similar or a little bit higher than assembly workers; hypertension in men aged in their 30s was 3%, which is similar or lower than for LCD workers.

When we compared lifestyle factors, the current smoking rate in semiconductor workers was 42%, and it is the same rate in the general population (42%). However, the smoking rate among subcontractor workers was 79%, which is more than the general population. Semiconductor workers reported that 22% of workers drink alcohol over 4 times a week. In the general population, 7.1% of those in their 30s and 7.2% of those aged in their 40s reported drinking alcohol over four times a week. In men, 8.8% in their 30s and 11.3% in their 40s and in women, 5.2% in their 30s drink alcohol over 4 times a week. Therefore, it seemed that semiconductor workers consumed higher levels of alcohol than the general population.

In the KNHANES, 5.9% of female workers over the age of 19 experienced a spontaneous abortion, and 6.5% of those with shiftwork [27]. However, this does not mean that a significant difference between the groups can be observed. In addition, infertility has been continuously reported by female semiconductor workers [28,29]. In this study, 6% of all female workers identified primary infertility. Based on the National Survey on Fertility Health and Welfare (2018), the fertility rate in women aged 15–39-year-old was 11.9%, about twice as high as that of semiconductor workers [30].

When we assessed the specific dietary habits in semiconductor workers, their rate of skipping meals was a little bit higher than that of the general population (Table S4). In the case of those aged in their 30s and 40s in South Korea, the rate of skipping meals was 31.6% (Male, 36.3%; Female, 26.7%) [25]. In particular, 74% of the operators and only 10% of the office workers skipped meals. As detailed dietary information, the intake rate was based on daily or one time per week, but it is limited to compare the frequency in the general population. Therefore, additional raw data analysis is needed in a further study based on KNHANES [31]. Although we cannot compare to other groups, vegetable and fruit intake rate in semiconductor workers was low (daily intake rate of vegetables in overall semiconductor workers was 5%; and intake of fruits was 1%). In addition, milk intake was at least 8-10 times higher than the general population because milk is regularly included in their diet. This suggests that a comprehensive dietary program considering vegetables and fruits is needed. Also, offering breakfast at work has been instituted since 2012 to provide balanced nutrients.

One of the purposes of this study was to assess the usefulness of the questionnaire and further use it in the cohort study on overall semiconductor workers. First, this questionnaire was constructed specializing in semiconductor workers which referred to the KNHANES and other national cohort studies. Through this, lifestyle status and others can compare to the Korean general population. In the case of individual disease history, questions were organized focusing on diseases that are mainly complained about by the semiconductor workers from previous studies or qualitative studies. Second, we assessed the response rate of the questionnaire. In this study, the non-responder in each question was lower than two participants (0.6% in the overall study population) and the overall non-response rate was lower than 5%. Third, we compared the concordance between the self-reported answer from the semiconductor workers and the answer when the interviewer asked the semiconductor workers. Thus, all of the answers were correct. Therefore, we concluded that this questionnaire is useful for semiconductor workers. In particular, the healthy worker effect should be considered in occupational epidemiology. First of all, defining the suitable comparison group to semiconductor workers is needed [32]. Previous studies, as comparison group office workers or the general population, were defined which could induce the healthy worker effects [33,34,35]. Therefore, under the semiconductor industry, LCD or assembly workers could be exposed to a low level of hazardous substances than fab or other workers. In addition, age at the time of employment, age at the time of exposure, and employment period affect the level of the healthy worker effect, so an appropriate sampling strategy is required [32]. Therefore, a prospective cohort study needs to be conducted to assess changes in employment status as a confounder.

As a limitation, identifying actual past occupational exposures and frequent diseases is limited because of individual opinions and the small number of volunteers in a qualitative study. In addition, factors such as radiation, magnetic exposure, and thermal decomposition products that are difficult to perceive for humans could not be assessed. Occupational or other risk factors were identified in the cross-sectional study, and information on environmental exposure was limited to the current exposure. Due to the selected PM engineers from the subcontractors, the distribution of factors and diseases may be deviated results compared to the total number of PM engineers. Even various strata of JEM were considered to select the study population, the possibility of misclassification could be existed due to the diverse semiconductor process. In addition, to fully assess the exact time of change in the semiconductor environment and cancer incidence considering their latent period, more workers who have long-term employment are needed. Nevertheless, this qualitative study confirmed the possibility that the past occupational exposure situation may be different from the present and the exposure intensity may be higher. It was also possible to list the diseases that are likely to occur in the special environment workers of fabrication. The cross-sectional study confirmed the acceptability of a questionnaire that could be used in future cohorts of semiconductor workers.

## 5. Conclusions

This study was the results of a qualitative survey based on 38 current and retired workers with at least 10 years of employment period and a cross-sectional pilot study from 306 current workers with at least 5 years of employment period. Diseases mainly reported in qualitative studies were also reported in the cross-sectional study, and through the information, we could identify common diseases under the current semiconductor environment. Compared to the general population, semiconductor workers’ medical, lifestyle, dietary behavior seemed to be similar or better. However, it cannot be generalized as a health effect on the overall semiconductor workers, so careful interpretation is needed. Also, the healthy worker effect should be considered for interpretation due to the fact that current workers comprised the majority of the study population. Additionally, based on the survey response rate, comparison of the response rate between the self-administrated questionnaire and the interviewed questionnaire, and non-concordant response rate, a semiconductor targeted questionnaire could apply in further prospective cohorts. Along with this, a prospective cohort design construction could establish a system that can check the risk signals of semiconductor workers through continuous health and exposure investigation and information construction. In advance of the construction of the cohort study, detailed improvement should be taken in the semiconductor environment such as in cleanroom garments and their specific patterns.

## Figures and Tables

**Table 1:** Demographic and occupational characteristics of semiconductor workers.

	Overall (306)	Office (24)	Assembly (43)	Operators (53)	LCD (38)	Non-Implant (59)	Implant (22)	PM (67)
	Med	Med	Med	Med	Med	Med	Med	Med
Age (year) *	36	45	40	37	32	39	35	35
Working hour/week	48	45	48	48	48	48	48	48
Working year *	13	21	20	17	7	7	12	11
Working day/month	23	21	23	24	23	24	24	22
Night working day/month	8	0	8	8	7	9	9	7
	%	%	%	%	%	%	%	%
Men *	72	50	51	0	100	100	100	100
Married *	66	92	89	68	50	58	64	58
Fab work (over 80% of work)	71	0	26	60	84	88	91	77
Among Fab workers								
Recognition of ‘Fab class management standards’	97		91	100	100	100	100	91
Shiftwork experiences								
Any shift works *	80	4	100	100	100	98	100	43
3-shifts 4-group #	73	0	100	98	73	39	36	24
2-shifts 3-group #	10	0	7	32	5	0	5	3
2-shifts 2-group	3	0	2	0	9	3	0	2
Shiftwork group at the survey day ^1^								
Day	24		37	32	30	33	32	2
Swing	25		33	38	34	34	27	6
GY	27		30	30	36	33	41	18
No shiftwork	24	100						74

Abbreviation: LCD, Liquid-crystal display; PM, Preventive Maintenance of semiconductor machines; Med, Median; Fab, Fabrication; GY, Graveyard. *, Statistically significant different among 7 groups, *p* < 0.05; Marginally significant, # 0.05 < *p* < 0.1. ^1^ Shiftwork hours in 3-shifts 4-group hours are defined as [Day] 06:00–14:00, [Swing] 14:00–22:00, and [GY] 22:00–06:00.

**Table 2:** Lifestyle factors, family history and personal history of diseases, and health supplement intake history for the past year in semiconductor workers.

	Overall (306)	Office (24)	Assembly (43)	Operators (53)	LCD (38)	Non-Implant (59)	Implant (22)	PM (67)
	%	%	%	%	%	%	%	%
Tobacco smoking								
Current smokers *	42	33	21	4	32	58	50	79
E-cigarette smokers #	6	4	0	2	2	8	0	18
Smoking ≥ 20 cigarettes a day *	10	62				15		5
Smokers ≥ 20 pack-year *	16	75	22	2	8	21	9	8
Alcohol consumption								
Drinking ≥ 4 times/week *	22	23	20	11	12	32	12	27
Alcohol intake ≥ 2.5 glasses/week*	46	41	32	32	50	52	27	57
Physical activity								
High-intensity *	16	4	9	2	24	31	14	18
Moderate-intensity *	40	8	35	28	68	56	50	31
Personal history of								
Gastritis or gastroduodenal ulcer ^1^	16	25	21	28	13	7	14	8
Dermatosis (allergic/contact) ^1^	9	8	7	19	8	8	0	6
Cystitis ^1^	6	8	9	13	5	2	0	3
Dry eye ^1^	7	8	7	13	5	8	0	1
Fatty liver ^2^	3	13	9	0	0	3	0	1
Hypertension ^2^	8	29	21	0	11	2	0	3
Hyperlipidemia ^2^	8	13	12	2	8	14	5	3
Liver disease (acute, chronic) ^2^	6	17	5	0	3	7	0	9
Diabetes ^2^	1	0	5	0	5	0	0	0
Periodontal disease	2	8	5	0	3	3	0	0
Intestinal polyps/polyps	2	17	0	0	0	2	5	1
Benign thyroid disease	2	4	5	0	0	2	0	1
Asthma	6	8	5	0	13	7	14	4
Angina pectoris ^2^	1	0	0	0	0	3	0	1
Lung tuberculosis	5	8	0	0	13	8	0	6
Nephrolithiasis	2	4	0	0	5	2	0	3
Comorbidity of personal diseases								
Two or more diseases	17	38	23	25	13	14	5	7
For fab-relating diseases	8	8	9	23	3	2	0	4
For cardiometabolic diseases	5	21	5	0	5	10	0	1
Other combinations	5	13	9	2	8	2	5	1
	**N**	**N**	**N**	**N**	**N**	**N**	**N**	**N**
Diagnosis of								
Malignant tumors or rare disease ^3^	5	1	2	1	1	0	0	0
	**M**	**M**	**M**	**M**	**M**	**M**	**M**	**M**
Body mass index (kg/m^2^)	24	23	24	22	26	24	23	24

Abbreviation: LCD, Liquid-crystal display; PM, Preventive Maintenance of semiconductor machines; Fab, Fabrication; M, Mean. *, Statistically significant different among 7 groups, *p* < 0.05; Marginally significant, # 0.05 < *p* < 0.1. ^1^ Fab-relating diseases: Groups of diseases and symptoms mentioned in the fab work group in a qualitative study. ^2^ Cardiometabolic diseases. ^3^ The cancers and rare diseases investigated in this survey are the same as those suggested in the list of diseases eligible for compensation for semiconductor workers employed by Samsung Electronics semiconductor and LCD.

**Table 3:** Health supplement intake history, hepatitis B vaccination history, experiences in medical procedures or specific imaging for a health check-up for the past year in semiconductor workers.

	Overall (306)	Office (24)	Assembly (43)	Operators (53)	LCD (38)	Non-Implant (59)	Implant (22)	PM (67)
	%	%	%	%	%	%	%	%
Taking for more than 3 months of								
Multivitamin *	19	46	9	15	20	29	23	7
Calcium supplement	2	21	2	2				
Korean ginseng supplement #	9	29	5	11	9	14	5	1
Low-dose aspirin	1	13		2				
Acetaminophen	2	8		6		2	5	
Vaccinated against hepatitis B ^1,^*	14	25	19	7	30	7	10	5
Medical procedures in the past year								
General anesthesia experience	14	21	19	12	15	7	17	10
Blood transfusion and acupuncture	15	29	16	16	15	18	12	
Radiation therapy experience	1	4			2			
Health check-up in the past year								
Gastroenterography *	22	20	30	6	40	14	20	9
Colonography	9	4	9	4	19	7	12	
CT scan *	43	54	51	25	34	61	47	41
MRI scan *	36	58	40	18	28	41	44	41
PET-CT examination	11	57	40		6			5
Nuclear medicine test	1	4						4

Abbreviation: LCD, Liquid-crystal display; PM, Preventive Maintenance of semiconductor machines; Fab, Fabrication. *, Statistically significant different among 7 groups, *p* < 0.05; Marginally significant, # 0.05 < *p* < 0.1. ^1^ Vaccinations near entering the company or since entering the company.

**Table 4:** Dietary habits for the past year in semiconductor workers.

	Overall (306)	Office (24)	Assembly (43)	Operators (53)	LCD (38)	Non-Implant (59)	Implant (22)	PM (67)
	%	%	%	%	%	%	%	%
Eating habit								
Eat salty	43	33	30	45	52	42	41	48
Eat fast meal < 10 min	29	33	23	36	36	24	27	28
Skipping meal								
1 or 2 times of 3 meals *	48	13	42	74	55	59	27	39
Breakfast ≥5 times a week *	39	17	21	55	30	31	19	27
Eating alone								
Breakfast *	37	75	16	11	39	36	64	46
Lunch	10	4	16	11	18	12	9	3
Dinner	16	8	5	11	20	19	27	21
Daily intake								
Fruit	1	4		2	2			
Vegetable	5	4	2	9	11	2	0	3
Soy product and soybean products	20	8	23	21	30	20	14	16
Mixed coffee *	24	0	40	20	16	10	9	31
Black coffee *	32	71	28	63	23	26	14	19
High sugar coffee #	5	13	2	2	16	3	0	2
Green tea	3	4	2	2	2	2	0	6
Intake at least once a week								
Milk #	46	42	44	34	61	48	32	52
Liquid yogurt	22	25	14	21	27	19	14	27
Yoghurt	13	17	14	11	11	10	18	13
Soda, Cokes	39	21	28	26	43	41	36	57
Fruit juice	26	25	14	17	32	29	18	36
Grilled Ham	22	8	19	15	21	25	14	34
Pizza	5	4	2	0	5	7	0	11
Hamburger/Sandwich	7	4	2	0	9	9	0	15

Abbreviation: LCD, Liquid-crystal display; PM, Preventive Maintenance of semiconductor machines; Fab, Fabrication. *, Statistically significant different among 7 groups, *p* < 0.05; Marginally significant, # 0.05 < *p* < 0.1.

**Table 5:** Blood markers of infections in semiconductor workers.

	Overall ^1^ (239)	Office (24)	Assembly (43)	Operators (53)	LCD (37)	Non-Implant (59)	Implant (22)
	%	%	%	%	%	%	%
HBV							
HBsAg + and HBsAb −	1	0	0	2	0	2	0
HBsAg + and HBsAb +	1	4	0	0	0	0	5
HBsAg − and HBsAb +	76	79	91	83	72	65	68
HBsAb − and HBsAg −	22	17	9	15	28	33	27
Anti-HCV +	0						
HIV	0.4						
*H. pylori*							
IgG +	33	50	33	40	26	26	32
IgG −	67	50	67	60	74	74	68
EBV							
VCA lgM +	5	4	0	9	5	2	14
VCA lgG +	97	100	91	100	95	97	100
EBNA lgG +	90	92	86	87	91	86	86

Abbreviation: LCD, Liquid-crystal display; PM, Preventive Maintenance of semiconductor machines; Fab, Fabrication. ^1^ In the human immunodeficiency virus (HIV-Ag/Ab combo) test and the T-cell leukemia virus antibody test, one each was confirmed to be positive: information on occupation was not classified to protect workers’ privacy. Maintenance and repair workers (67) and LCD production workers (1) were excluded because blood collection was not performed.

## Data Availability

No new data were created or analyzed in this study. Data sharing is not applicable to this article.

## Supplementary Materials

The following supporting information can be downloaded at: https://www.mdpi.com/article/10.3390/ijerph19106286/s1, Table S1: Main questions and answers of study subjects in a qualitative study on past work ex-posure and health status of Samsung semiconductor workers; Table S2: Sampling strata and algorithms for selection of subjects in a qualitative study on past work exposure and health status of Samsung semiconductor workers; Table S3: Sampling strata and algorithms for the selection of subjects in a pilot study on current work exposure and health status of Samsung semiconductor workers; Table S4: List of compensatory diseases for semiconductor workers employed in Samsung Electronics Semiconductor and LCD; Table S5: History of reproductive factors, abortion, and infertility in 86 female semiconductor workers. Figure S1: Hierarchy of study population selection.
