# Supplementary material for: Health Status, Health-Related Factors and Work Environment in Korean Semiconductor Workers between 1984–2012: A Qualitative Study and a Cross-Sectional Study
Source: Int J Environ Res Public Health. 2022 May 22;19(10):6286. doi: 10.3390/ijerph19106286 (PMC9140394; doi:10.3390/ijerph19106286)
Supplement: Supplementary file 1 [file ijerph-19-06286-s001.zip › ijerph-1697405-supplementary.pdf]

International Journal of
Environmental Research and Public Health

Table S1. Main questions and answers of study subjects in a qualitative study on past work exposure and health status of Samsung semiconductor workers.

	Contents
Question 1	What was the environment in which workers worked in the past? How is it different from the present?<br>(Example: Did you do the semiconductor process automatically, semi-automatically, or manually)
**Answers**	o In the past, the physical burden was reduced by introducing a robot (AGV) to move between processes, although in the past, human resources were used separately due to strain on the body.<br>o Currently, the line is automated and the physical burden was reduced.<br>o In the past, wafers were moved one by one by hand, but now, all transfers between processes are automated.<br>o In the past, workers' awareness of environmental safety was low; but currently, workers' awareness of environmental safety was good.<br>o Compared to the past, education on environmental safety and chemicals has been strengthened.<br>o In the past, the standards for wearing personal protective equipment have been strengthened.<br>o In the past, there were insufficient safety precautions or guidelines on how to operate.<br>o In the past, when equipment broke down, it was simply necessary to turn it on again, but now there are many preventive activities to avoid repeating why it happened when it broke down.
Question 2	Have you ever been exposed to odors, air quality, or environmental exposure substances in the past and present working environments?
Answers	[Sub-topic derived from interview contents]<br>1. Past situation<br>o In the past, there was a possibility of exposure to hazardous substances, but the current semiconductor production process is automated and exposure to hazardous substances is rare.<br>o In the past, some workers may not be unaware of the harmfulness of chemicals and in some cases handled small amounts.<br>o At the time I was working, I honestly didn't know that it was harmful. After some time has passed and looked back now, it is a chemical.<br>o TCE.<br>- In the past, we used TCE to wipe oil in hands and equipment and to clean using TCE because it was very effective at wiping off any grease or anything on it.<br>- We used it without any restrictions because TCE was written as no restriction for use that way in the "Instructions for use". After a few years, it was replaced.<br>o Plating and marking processes in one space without distinction:<br>- In the past, plating and marking were done in one space. We worked while inhaling white chemic al smoke and odors when plating.<br>- A guy in the same plating process was diagnosed with lymphoma later.<br>2. Current situation<br>o Currently, most processes are automated and there is no exposure to hazardous substances.<br>o It is not loud in semiconductor devices, but it makes a little noise.<br>o There is a slight chemical smell in the machine room that supplies chemicals in the current CCSS room.<br>o Risk of being pierced by glass fragments for those working in glass shredding (1 person).<br>o Chemical detection sensor<br>- The chemical detection sensor can prevent to exposure of ionizers or the fumes that come out whe n the PR is processed at a high temperature.<br>- Current processes were as follows: The wafer goes into the equipment, and in the equipment, the wafer goes into the chemicals. And then the wafer comes out after washing it with water and dryi ng it in the same equipment.<br>- There is no leaking from the inside out. The sensor detects the leaking of equipment if there is. T here are also sensors to detect the leaking chemicals. The equipment sucks out fumes and gases wi th exhaust. When the pressure drops, the sensor in the equipment was alarmed and the equipment stop.
Question 3	How was your shift work? (Please explain if it is different from what is indicated on the questionnaire). Isn't shift work difficult?
Answers	Sub-topic derived from interview contents<br>o It has been confirmed that the type of shift work is consistent with that described in the questionnaire.<br>o Shift work is currently 4 teams and 3 shifts, but in the past, it was slightly different, so there were times when 3 teams and 3 shifts or 2 shifts were used.

	o Most workers said that work shift is difficult due to biorhythm changes such as lifestyle changes, difficulty till adaptation next shifting schedule, irregular sleep, and eating, etc.<br>o Once you get used to the three-shift schedule, there are not many difficulties. They stated to be generally satisfied with the current working environment.
Question 4	If there have been any clusters of symptoms or diseases that have occurred to you and your co-workers from the past to the present, please tell me as much as you can remember.
Answers	[Sub-topic for common symptoms derived from interview contents]<br>o Pain and musculoskeletal disorders such as wrist, back, and knee used when lifting objects<br>o Eczema caused by wearing gloves<br>o Ear abnormality due to mechanical noise sound (1 person)<br>o Poor eyesight due to yellow light and dry eyes<br>o Currently, there is no experience of onset or symptoms of disease due to exposure to harmful substanc es.<br>o Past diagnosis: Lymphoma (in a beneficiary retiree)

TCE, Trichroloethylen; CCSE, Central chemical supply system.

Table S2. Sampling strata and algorithms for selection of subjects in a qualitative study on past work exposure and health status of Samsung semiconductor workers.

Criteria	Contents																	
1. 1st criterion	Current workers														Subcontractor [1]		Retired Workers	
2. District	Giheung				Giheung				Asan		Onyang				GiheungNA			
3. Fab. & Line	Non-Fab.				Fab.: 8-inch line				Fab.: LCD line		Assembly/packages				NA		NA	
4. Duty	Office workers				Operator		Process or mechanical engineers		Process or mechanical engineers		Assembly workers Process or mechanical engineers				PM		Operator	Process or mechanical engineers
5. Process	NA				NA		SC process (8): Diffusion, Cleaning, Photo, Etching, Implant, CVD, Metal, CMP		LCD process (7): TFT-Diffusion, TFT-Photo, TFT-Etching, CF-ITO/BM, CF-RGB, Cell-Conjugation & Cutting Cell-LC injection		NA				NA		NA	
6. Sex	Men		Women		Women		Men		Men		Men		Women		Men		Women	Men
7. Employment period (years)	≥15	10–14	≥15	10–14	≥15	10–14	≥15	10–14	≥15	10–14	≥15	10–14	≥15	10–14	≥15	10–14	≥15	10–14
Strata, n	1	1	1	1	1	1	16	16	14	14	1	1	1	1	1	1	1	1
Eligible workers with meeting strata conditions, n	894	643 [2]	155	220 [2]	205	402 [2]	55	97	9	154	184	534 [2]	301	741 [2]	NA	NA	NA	NA
A random sampling of candidate workers to be contacted for study participation was conducted from a pool of eligible workers.															Non-random sampling			
Strata selected for random sampling, n	1	0	1	0	1	0	16	16	14	14	1	0	1	0	1	1	1	1
Workers agreed to participate in the study																		
Final participants, n	3	0	3	0	4	0	2	7	1	6	3	0	3	0	0 [3]	3	2	1 [3]
Sampling fraction, %	0.6%				2.0%		5.9%		5.5%		1.2%				75%		75%	

Abbreviation: NA, Not applicable; Fab, Fabrication; LCD, Liquid-crystal display; PM, Preventive Maintenance of semiconductor machines; CVD, Chemical vapor deposition; CMP, Chemical mechanical polishing, TFT, Thin-film-transistor; CF, Color filter; Ito, Indium tin oxide; BM, Black Matrix; LC, Liquid-crystal; RGB, Red-green-blue; LC, Liquid-crystal. [1]. Retirees who could be contacted were invited to the study, and workers from outsourcing companies who responded that workers could participate were invited to participate in the study. [2]. The category of workers with 10-14 years of service was not used in the random sampling because the number of eligible workers with more than 15 years of service was sufficient. [3]. One retiree did not participate in the study.

Table S3. Sampling strata and algorithms for the selection of subjects in a pilot study on current work exposure and health status of Samsung semiconductor workers.

Criteria									
1. 1st criterion	Current workers								Subcontractor [1]
2. District	Giheung<br>Hwaseong		Giheung<br>Hwaseong<br>Asan	Giheung<br>Hwaseong		Asan	Onyang		Giheung<br>Hwaseong
3. Fab. & Line	Non-Fab.		Fab	8-inch Fab	16-inch Fab	Fab.: LCD line	Assembly/packages		NA
4. Duty	Office workers		Operator	Process or mechanical engineers		Process or mechanical engineers	Assembly workers<br>Process or mechanical engineers		PM
5. Process				SC process (6): Diffusion, Cleaning, Photo, CVD, Metal, CMP	SC process (2): Etching, Implant	LCD process (5): TFT-Diffusion, TFT-Photo, TFT-Etching, CF-ITO/BM, CF-RGB,<br><br>* Excluded: Cell-Conjugation & Cutting Cell-LC injection			Implant, Etching
6. Sex	Men	Women	Women	Men	Men	Men	Men	Women	Men
7. Employment period (years)	Eligible workers with meeting strata conditions, n								
≥15	1946	351		67	29	153	48	223	
5–14	1825	562		721	218	463	385	659	
≥10			409						125
<10									471
	Final participants, n								
	Office (12)	Office (12)	Operator (53)	SC non-implant (59)	Implant (22)	LCD (38)	Assembly (43)		PM (67)
≥15	8	8		2	2	12	11	11	
5–14	4	4		57	20	26	11	10	
≥10			53						44
<10									23
Participation rate, %	0.5%		11.5%	7.5%	8.9%	6.2%	3.3%		11.2%

Abbreviation: NA, Not applicable; Fab, Fabrication; LCD, Liquid-crystal display; PM, Preventive Maintenance of semiconductor machines; CVD, Chemical vapor deposition; CMP, Chemical mechanical polishing, TFT, Thin-film-transistor; CF, Color filter; Ito, Indium tin oxide; BM, Black Matrix; LC, Liquid-crystal; RGB, Red-green-blue; LC, Liquid-crystal. [1]. Out of all outsourced partners, only those outsourcing companies that allow workers to participate are selected. Of workers from outsourcing companies who responded that workers could participate, those who worked on the day of the survey participated in the study.

Table S4. List of compensatory diseases for semiconductor workers employed in Samsung Electronics Semiconductor and LCD [1.]

Group	Disease Name	ICD-10 Disease Code
Cancer	Leukemia	C91~C95
	Non-Hodgkin lymphoma	C82~C86, C96.7, C96.9
	Multiple myeloma and malignant plasma cell neoplasm	C90
	Myelodysplastic syndrome	D46
	Other aplastic anemias and other bone marrow failure syndromes	D61
	Other neoplasms of uncertain behavior of lymphoid, hematopoietic and related tissue	D47
	Extranodal marginal zone B-cell lymphoma of mucosa-associated lymphoid tissue [MALT-lymphoma]	C88.4
	Malignant neoplasm of brain and central nervous system	C70, C71, C72(C72.2- C72.9), C75(C75.1-C75.3)
	Malignant neoplasm of larynx	C32
	Malignant neoplasm of trachea, bronchus and lung	C33, C34
	Malignant melanoma of skin	C43
	Malignant neoplasm of ovary	C56
	Malignant neoplasm of breast	D05,C50
	Malignant neoplasm of prostate	C61
	Malignant neoplasm of rectum	C20
	Malignant neoplasm of pancreas	C25
	Benign neoplasm of brain and central nervous system	D32, D33(D33.0- D33.3), D35(D35.2-D35.4), D42, D43(D43.0-D43.3), D44(D44.3-D44.5)
	Malignant neoplasm of kidney, except renal pelvis	C64
	Malignant neoplasm of stomach (Female only)	C16
	Malignant neoplasm of eye and adnexa	C69
	Malignant neoplasm of nasal cavity and middle ear	C30
	Malignant neoplasm of accessory sinuses	C31
	Malignant neoplasm of parotid gland	C07
	Malignant neoplasm of other and unspecified major salivary glands	C08
	Malignant neoplasm of nasopharynx	C11
	Malignant neoplasm of peripheral nerves of thorax	C47.3
	Malignant neoplasm of lip	C00
	Malignant neoplasm of oropharynx	C10
	Malignant neoplasm of other and ill-defined sites in the lip, oral cavity and pharynx	C14
	Malignant neoplasm of anus and anal canal	C21
	Malignant neoplasm of vulva	C51
	Malignant neoplasm of vagina	C52
	Malignant neoplasm of uterus, part unspecified	C55
	Malignant neoplasm of other and unspecified female genital organs	C57
	Malignant neoplasm of placenta	C58
	Malignant neoplasm of penis	C60
	Malignant neoplasm of other and unspecified male genital organs	C63
	Malignant neoplasm of other and unspecified urinary organs	C68
	Malignant neoplasm of adrenal gland	C74
	Malignant immunoproliferative diseases and certain other B-cell lymphomas	C88
Rare diseases	Multiple sclerosis	G35
	Sicca syndrome [Sjögren]	M35.0
	Systemic sclerosis [scleroderma]	M34
	Amyotrophic lateral sclerosis	G12.21
	Wegener's granulomatosis	M31.3
	Systemic lupus erythematosus (SLE)	M32

	Rare intractable disease (Patients with Idiopathic pulmonary fibrosis assigned to this code receive medical cost reductions from the Korea National Health Insurance Service (K-NHIS) by up to 10% of the total cost)	J84.18
	Parkinson's disease and Parkinsonism: primary, secondary, and classified elsewhere	G20-G22
	Adult-onset Still's disease	M06.1
	Immune thrombocytopenic purpura	D69.3
Diseases for children of workers	Congenital malformations, deformations and chromosomal abnormalities	Q00-Q99
	Malignant neoplasm	C00-C97
	Polycythemia vera	D45
	Myelodysplastic syndromes	D46
	Other neoplasms of uncertain behavior of lymphoid, hematopoietic and related tissue	D47
	Acquired pure red cell aplasia [erythroblastopenia]	D60
	Other aplastic anemias and other bone marrow failure syndromes	D61
	Rare diseases for children of workers (The scope of rare diseases is based on the list of rare diseases designated for national management by the Ministry of Health and Welfare.)	-
Reproductive disease	Recurrent pregnancy loss	N96
	Hydatidiform mole	O01
	Other abnormal products of conception	O02
	Spontaneous abortion	O03
	Complications following (induced) termination of pregnancy	O04
	Other abortion	O05
	Unspecified abortion	O06
	Maternal care for intrauterine death	O36.4
	Stillbirth	P95
	Single stillbirth	Z37.1
	Twins, one liveborn and one stillborn	Z37.3
	Twins, both stillborn	Z37.4
	Other multiple births, some liveborn	Z37.6
	Other multiple births, all stillborn	Z37.7
	Continuing pregnancy after spontaneous abortion of one fetus or more	O31.1
	Continuing pregnancy after intrauterine death of one fetus or more	O31.2

[1]. Information about the Criteria for selection of beneficiaries of compensation, the amount of compensation, and a detailed list of compensatory diseases can be found in the website of "Support and compensation for occupational health and welfare of Samsung Electronics semiconductor and LCD workers" (http://www.ohsec.or.kr/rewardSystem/compensability.asp, accessed on 20 May 2022).

Table S5. History of reproductive factors, abortion, and infertility in 86 female semiconductor workers.

	Women<br>N = 86
	Median
Age at menarche (years)	15
	N
Mensturation cycle	
Irregular [1]	14
Regular	72
Pregnancy history	
Never pregnant [2]	32
1–2	44
3–4	10
Spontaneous abortion [3]	
0	46
1	5
≥2	3
Spontaneous abortion in 1st pregnancy [4]	6
Testing history for amniotic fluid, chorion, or chromosomes	
Obstetricians have ever recommended a test for amniotic fluid, chorion, or chromosomes during pregnancy.	9
I was actually tested.	7
My test result was abnormal	0
Experience diagnosed with infertility [5]	
No experience of infertility	70
Primary infertility	5
Secondary infertility	11
Experience with infertility treatment [6]	16
Causes of infertility [6]	
Female worker's problem	2
Female worker + her spouse	1
Spouse's problem	2
Unknown	11

[1]. Average menstrual cycle (<21 days or >35 days). [2]. All 6 LCD production workers had no experience of pregnancy. [3]. Among women who have been pregnant (N = 54). [4]. Among women who have been spontaneous abortion (N = 8). [5]. Primary infertility was defined as the infertility in women who have never been pregnant; Secondary infertility was defined as the infertility in women who have been pregnant. 6. Among female workers who have experienced infertility (N = 16).

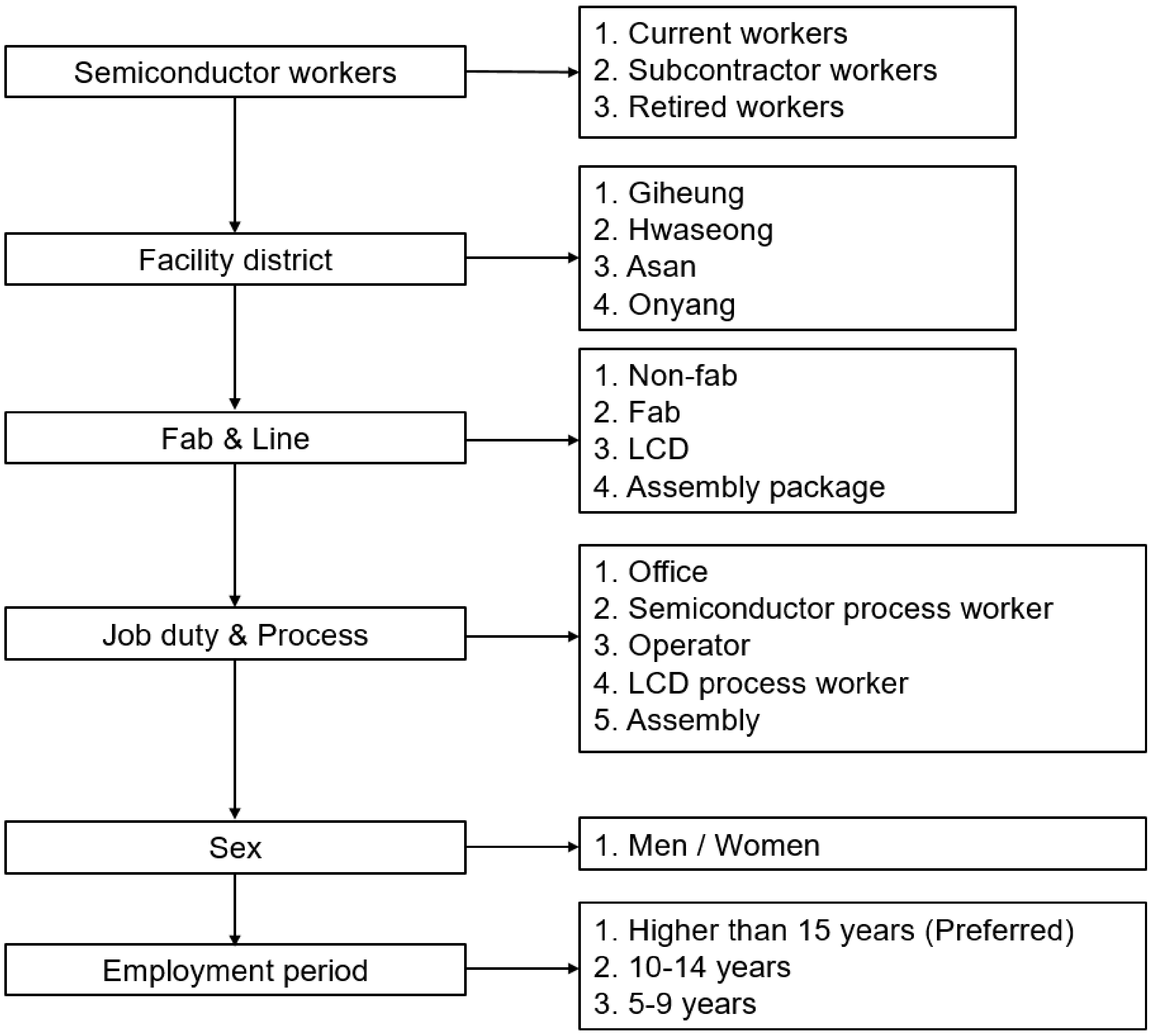

Figure S1. Hierarchy of study population selection.
